# Dopaminergic neuronal dysfunction induced by newer generation systemic insecticides in *Caenorhabditis elegans*

**DOI:** 10.64898/2026.06.14.732178

**Published:** 2026-06-18

**Authors:** Adam R Filipowicz, Keane Bui, Nada Osman, Katherine S Morton, Isabel W Kenny-Ganzert, David R Sherwood, Joel N Meyer, Patrick Allard

## Abstract

While a growing number of studies have linked environmental exposures and Parkinson’s disease (PD) ^1–3^ , the impact of many pesticides remains understudied ^4,5^ ; for example, neonicotinoids are the most used insecticides in the world, but research into their contribution to PD is limited to a handful of studies ^6–8^ . Newer pesticides, such as the butenolide flupyradifurone (FPF), specifically developed to overcome increased pest resistance ^9^ and spurred on by tighter restrictions on neonicotinoids such as imidacloprid (IMI) ^10^ , are even less studied. New approach methodologies (NAMs) that allow for rapid evaluation of pesticide exposures are needed to evaluate potential links between the growing number of pesticides and PD ^11^ . To this end, we exposed the model nematode *Caenorhabditis elegans* ^12^ to IMI and FPF. Due to its high degree of tractability, and conservation of many genetic, neuronal, and toxic mode of action processes, *C. elegans* has been invaluable in both elucidating mechanisms and novel therapeutic targets for PD that can be validated in other models ^13^ , and as a complementary tool for early toxicity screening ^14^ . Along this line, we found that exposure to IMI, and to a greater extent FPF, in young adult animals causes significant dendritic blebbing, an early sign of neurodegeneration, exclusively in dopaminergic neurons. Blebbing was accompanied by impairment of dopamine-mediated behaviors, changes in neuronal mitochondrial morphology, and elevation of pathways related to reactive oxygen species (ROS). We were able to reduce the blebbing caused by IMI and FPF two ways: 1) pharmacologically via administration of the antioxidant N-acetyl cysteine (NAC); 2) genetically via knockout of a MAP kinase (MAPK) stress response pathway. This suggests that oxidative stress is a key mediator of this insecticide-induced dopaminergic neurodegeneration.

## Full Text Availability

The license terms selected by the author(s) for this preprint version do not permit archiving in PMC. The full text is available from the preprint server.

